# Loss of the Acetate Switch in Vibrio vulnificus Enhances Predation Defense against Tetrahymena pyriformis

**DOI:** 10.1128/AEM.01665-21

**Published:** 2022-01-25

**Authors:** Viduthalai Rasheedkhan Regina, Parisa Noorian, Clarence Bo Wen Sim, Florentin Constancias, Eganathan Kaliyamoorthy, Sean C. Booth, Gustavo Espinoza-Vergara, Scott A. Rice, Diane McDougald

**Affiliations:** a Singapore Centre for Environmental Life Sciences Engineering, Nanyang Technological University, Singapore; b The iThree Institute, University of Technology Sydney, Sydney, Australia; c The School of Biological Sciences, Nanyang Technological University, Singapore; Royal Netherlands Institute for Sea Research

**Keywords:** protozoan predation, grazing resistance, overflow metabolism, acetate switch, predation defense, *Vibrio vulnificus*

## Abstract

Vibrio vulnificus is an opportunistic human pathogen and autochthonous inhabitant of coastal marine environments, where the bacterium is under constant predation by heterotrophic protists or protozoans. As a result of this selection pressure, genetic variants with antipredation mechanisms are selected for and persist in the environment. Such natural variants may also be pathogenic to animal or human hosts, making it important to understand these defense mechanisms. To identify antipredator strategies, 13 V. vulnificus strains of different genotypes isolated from diverse environments were exposed to predation by the ciliated protozoan Tetrahymena pyriformis, and only strain ENV1 was resistant to predation. Further investigation of the cell-free supernatant showed that ENV1 acidifies the environment by the excretion of organic acids, which are toxic to T. pyriformis. As this predation resistance was dependent on the availability of iron, transcriptomes of V. vulnificus in iron-replete and iron-deplete conditions were compared. This analysis revealed that ENV1 ferments pyruvate and the resultant acetyl-CoA leads to acetate synthesis under aerobic conditions, a hallmark of overflow metabolism. The anaerobic respiration global regulator *arcA* was upregulated when iron was available. An Δ*arcA* deletion mutant of ENV1 accumulated less acetate and, importantly, was sensitive to grazing by *T. pyriformis*. Based on the transcriptome response and quantification of metabolites, we conclude that ENV1 has adapted to overflow metabolism and has lost a control switch that shifts metabolism from acetate excretion to acetate assimilation, enabling it to excrete acetate continuously. We show that overflow metabolism and the acetate switch contribute to prey-predator interactions.

**IMPORTANCE** Bacteria in the environment, including *Vibrio* spp., interact with protozoan predators. To defend against predation, bacteria evolve antipredator mechanisms ranging from changing morphology, biofilm formation, and secretion of toxins or virulence factors. Some of these adaptations may result in strains that are pathogenic to humans. Therefore, it is important to study predator defense strategies of environmental bacteria. V. vulnificus thrives in coastal waters and infects humans. Very little is known about the defense mechanisms V. vulnificus expresses against predation. Here, we show that a V. vulnificus strain (ENV1) has rewired the central carbon metabolism, enabling the production of excess organic acid that is toxic to the protozoan predator *T. pyriformis*. This is a previously unknown mechanism of predation defense that protects against protozoan predators.

## INTRODUCTION

Vibrio vulnificus is a Gram-negative, halophilic bacterium that thrives in warm marine and estuarine waters. Despite its environmental origin, the bacterium is associated with opportunistic infections that include gastrointestinal infections caused by ingestion of raw or undercooked seafood as well as wound infections caused by exposure of wounds or broken skin to estuarine water or seawater resulting in sepsis (1). V. vulnificus is responsible for the highest fatality rate among foodborne pathogens (1, 2) and exhibits considerable genetic and phenotypic variation. An allelic difference in a virulence-correlated gene locus (*vcg*) distinguishes the environmental (e.g., from oysters, clams, shrimp, seawater, and sediment) E-genotype strains (*vcgE*) from the clinical genotype strains (*vcgC*) (3, 4). More recent analysis of V. vulnificus genomes reveals that there are four major clusters (5) or five lineages (6) of strains. The strain studied here, ENV1, belongs to cluster 2, lineage 2. The emergence and persistence of pathogenic strains from the environment are attributed in part to evolutionary adaptations for protection against predation by protozoans (7–9). Bacteria possess multiple predator defense strategies, including extracellular defenses to avoid ingestion and intracellular defenses that include toxin secretion, that are ascribed to the origins of extracellular and intracellular pathogenesis (10).

To avoid predation, bacteria such as Comamonas acidovorans (11) and *Flectobacillus* spp. (12) form filaments under predation pressure while Salmonella spp. (13) alter surface antigens. Biofilm formation is another common strategy for resisting predation (14). In addition to predator avoidance, some bacteria kill and lyse the predator, which has the additional advantage of providing the pathogen with additional nutrients from dead predators (15). Vibrio cholerae, Vibrio fischeri, Janthinobacterium lividum, and Chromobacterium violaceum are some of the bacteria that excrete extracellular toxic factors to kill the protozoans (16–19). Escherichia coli also produces toxins, such as Shiga toxin (Stx), that can kill Tetrahymena thermophila (20). In V. cholerae, the type VI secretion system enables contact killing of the amoeba Dictyostelium discoideum and also affects mammalian macrophages (21–23). The PrtV protein kills the flagellate Cafeteria roenbergensis and the ciliate Tetrahymena pyriformis (24). Furthermore, release of reactive oxygen species, quorum sensing-mediated biofilm formation, production of vibrio polysaccharides (VPS), and the chitin-dependent production of ammonia (14, 19, 25, 26) are additional mechanisms that V. cholerae uses to kill protozoan predators. In contrast, the predator defense mechanisms of V. vulnificus are not well studied. One well-known predator defense factor of V. vulnificus is the multifunctional-autoprocessing repeats-in-toxin (MARTX), encoded by the *rtxA1* gene, that causes plasmolysis of amoebae (27). MARTXs kill cells by forming pores in the cell membranes (28). However, with the exception of MARTXs, no other mechanisms for predation resistance in V. vulnificus have been characterized.

The aim of this study was to identify the grazing defense mechanisms of an environmental strain of V. vulnificus, ENV1. By analysis of the cell-free supernatants of ENV1, transcriptomic analysis, and quantification of the excreted metabolites, we show that ENV1 has adapted to overflow metabolism by fermenting pyruvate to acetate, despite the presence of oxygen. Overflow metabolism, coupled with the loss of acetate assimilation, creates an acetic acid-rich environment that is toxic to *T. pyriformis*. We propose that overflow metabolism and the acetate switch are novel antipredator mechanisms of V. vulnificus ENV1.

## RESULTS

### V. vulnificus ENV1 is resistant to *T. pyriformis* predation.

**FIG 1:**
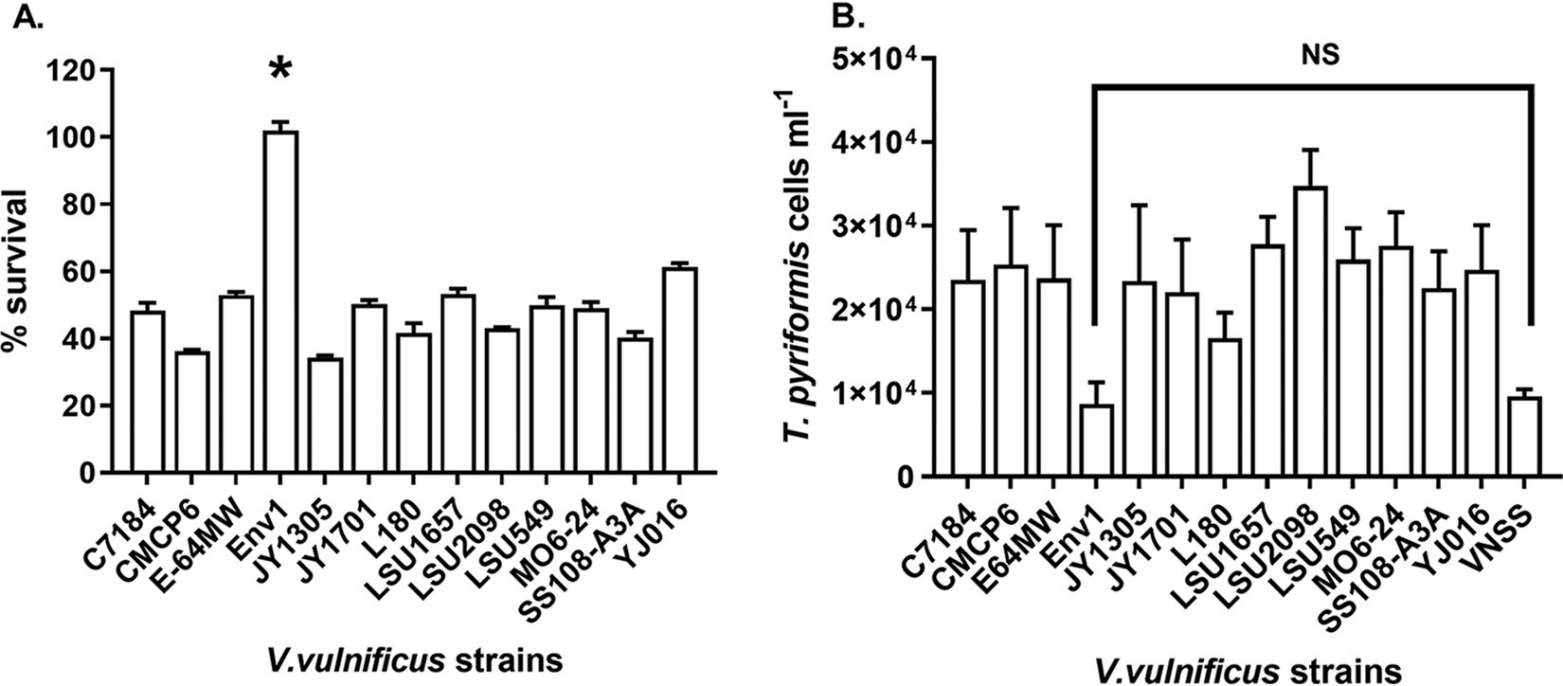
Percentage of bacterial survival (A) and number of *T. pyriformis* cells (B) after 24 h of coculture between different strains of V. vulnificus with *T. pyriformis.* The percentage of V. vulnificus survival was quantified by measuring optical density of the coculture (OD _600_) in relation to the control cells grown without *T. pyriformis* (100% survival) (A). The number of *T. pyriformis* cells was determined from the coculture by direct counts using an inverted microscope under bright field illumination and compared with the cells cultured without bacteria (B). Experiments were conducted with three replicate samples and repeated three times separately. Error bars represent standard deviation. Statistical analysis was performed using two-way ANOVA (A) and one-way ANOVA and Dunnett’s multiple-comparison test comparing all strains with the VNSS media control (B). *, *P* < 0.0001 (note: cell densities of ENV1 grown with and without *T. pyriformis* were similar) (A); NS, *P *> 0.05 (B).

Thirteen strains of V. vulnificus, representing different genotypes and isolation sources (Table 1), were assessed for grazing resistance against T. pyriformis. Twelve strains showed significant reduction in bacterial biomass compared to that of the bacteria grown alone (Fig. 1A), and the number of *T. pyriformis* cells also increased significantly compared to that of cells grown without bacteria (Väätänen nine-salt solution [VNSS] control) (Fig. 1B). In contrast, the biomass of strain ENV1 was not reduced by *T. pyriformis* predation (Fig. 1A) and the number of *T. pyriformis* cells was not significantly different from that of the control (Fig. 1B). This suggests that, in contrast to the other 12 strains, ENV1 defended against predation by *T. pyriformis*. The toxicity of ENV1 cell-free supernatants (CFS) was also tested on the saltwater ciliate, Uronema marinum. The addition of ENV1 CFS caused the same cell death observed in *T. pyriformis* after less than 5 min (data not shown).

**TABLE 1:** List of bacterial and protozoal strains

Strain	Properties	Source^*a*^	Reference
Bacteria			
CMCP6	C-genotype, clinical isolate, WT	Human blood (South Korea)	60
C7184	C-genotype, clinical isolate, WT	Human blood (Atlanta)	61
MO6-24	C-genotype, clinical isolate, WT	Human blood (California)	62
YJ016	C-genotype, clinical isolate, WT	Human blood (Taiwan)	63
L-180	C-genotype, clinical isolate, WT	Human blood (Japan)	64
JY1701	E-genotype, environmental isolate, WT	Oyster (Louisiana)	3
JY1305	E-genotype, environmental isolate, WT	Oyster (Louisiana)	3
ENV1	E-genotype, environmental isolate, WT	Oyster (Louisiana)	3
SS108-A3A	E-genotype, environmental isolate, WT	Oyster (Louisiana)	3
LSU2098	E-genotype, clinical isolate, WT	Human wound (Nk)	3
LSU549	E-genotype, clinical isolate, WT	Human wound (Nk)	3
LSU1657	E-genotype, clinical isolate, WT	Human wound (Nk)	3
E64MW	E-genotype, clinical isolate, WT	Human wound (Nk)	3
Protozoa			
*T. pyriformis*	Wild type		ATCC 205063
*U. marinum* (Dujardin 1841)	Wild type		Isolated by Martina Erken (2011, Sydney Institute of Marine Science)

a Nk, not known.

### V. vulnificus ENV1 excretes a pH-sensitive toxic factor in the presence of iron.

**FIG 2:**
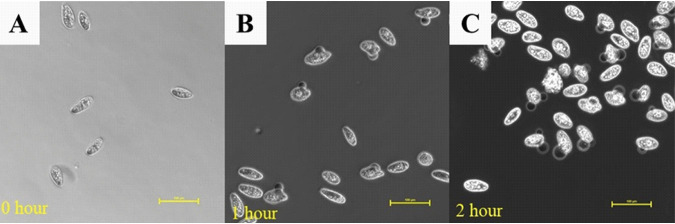
Visualization of *T. pyriformis* incubated in CFS of V. vulnificus ENV1 over 2 h. Actively swimming cells (A) stopped swimming and sank to the bottom of the plate, and cells appear to leak cytoplasm after 1 h of incubation (B). Complete lysis and degradation of cell membranes was observed after 2 h (C). Scale bar = 100 μm.

To determine if ENV1 defense was based on secreted factors, we incubated *T. pyriformis* with the CFS of ENV1 grown for 24 h in 0.5× VNSS medium. After 10 min at room temperature, the ciliates stopped swimming and sank to the bottom of the microtiter plates. After 1 h, the ciliates were dead (100%) with their cytoplasm leaking out (Fig. 2B). After 2 h, the ciliate cells were observed to be degraded (Fig. 2C). In contrast, *T. pyriformis* incubated in VNSS without CFS remained healthy throughout the experiment (Fig. 2A). This effect of the ENV1 CFS on *T. pyriformis* suggested that the bacteria excrete toxic factors that can permeabilize and degrade the cell membrane of *T. pyriformis*, leading to cell death.

**FIG 3:**
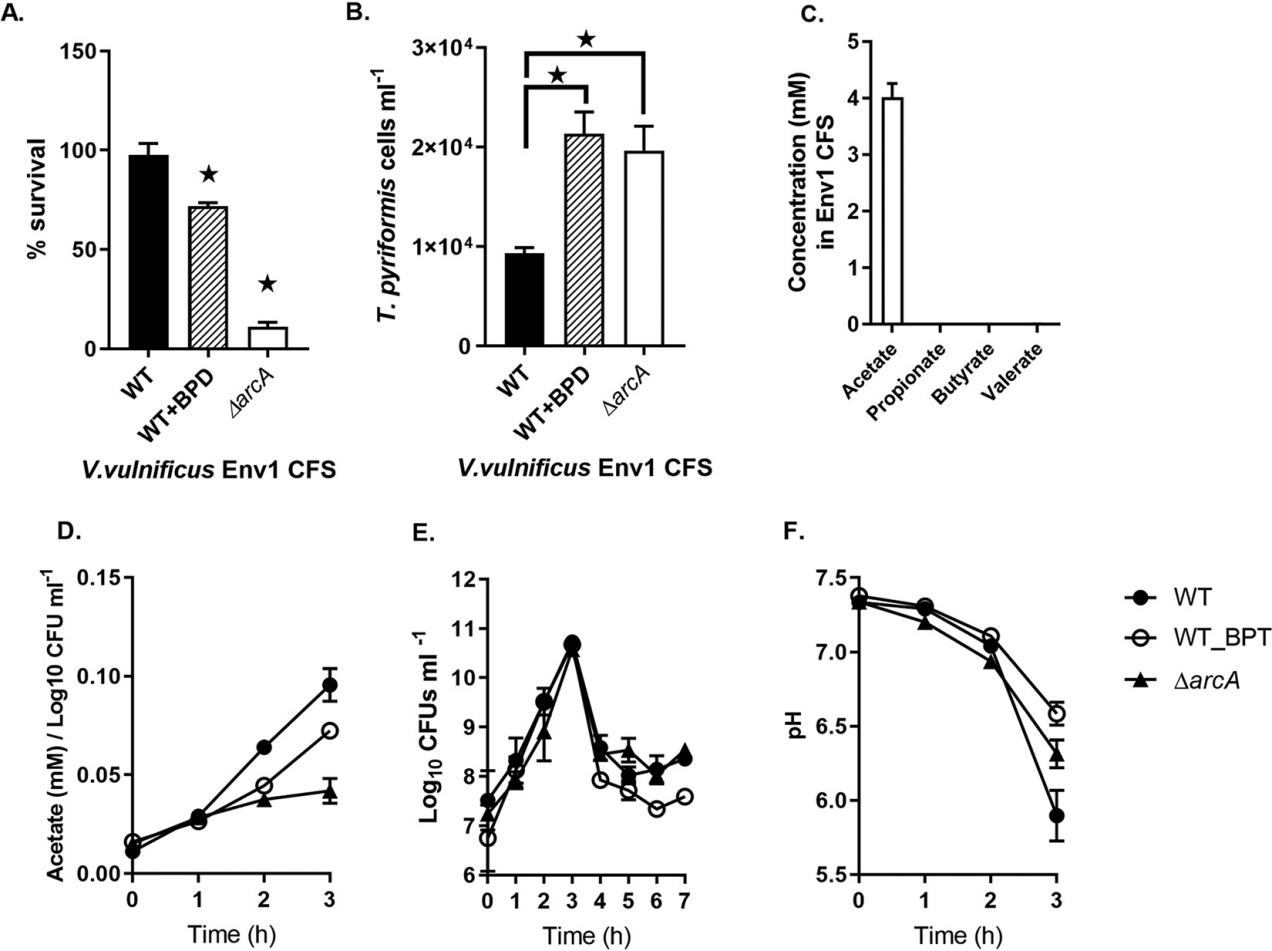
Percentage of bacterial survival (A) and number of *T. pyriformis* cells (B) after 24 h of coculture of V. vulnificus ENV1 WT, ENV1 WT supplemented with 2-2′ bipyridyl (BPD), and Δ*arcA* mutant with *T. pyriformis*. Concentration of short-chain fatty acids (acetate, propionate, butyrate, and valerate) in the CFS of ENV1 after 24 h of growth under aerobic conditions at RT (C). Acetate excretion (D), growth at OD_600_ (E), and pH (F) of V. vulnificus ENV1 WT (filled circles), ENV1 WT supplemented with 2-2′ bipyridyl (open circles), and Δ*arcA* (filled rectangles). Growth was measured up to 7 h, and acetate concentration and pH were measured up to 3 h where growth was maximal. Experiments were run in 0.5× VNSS medium with at least 3 replicates. Error bars represent standard deviation. Statistical analysis was performed using 2-way ANOVA and Sidak’s multiple-comparison test (A) and one-way ANOVA and Dunnett’s multiple-comparison test comparing all strains with the VNSS media control (B). Star, *P* < 0.05.

V. vulnificus is a ferrophilic bacterium that requires high levels of iron for pathogenicity (29). Therefore, the role of iron in grazing resistance was evaluated by assessing the toxicity of CFS of ENV1 grown under iron-depleted conditions (by supplementing the 0.5× VNSS with 2-2′ bipyridyl). The CFS produced under iron-depleted conditions was unable to kill *T. pyriformis* (Table 2). The iron limitation enabled grazing on ENV1 (Fig. 3A), and the number of *T. pyriformis* doubled (Fig. 3B). The loss of toxicity toward *T. pyriformis*, and the loss of grazing resistance of ENV1 when grown under iron-depleted conditions, strongly suggests that excretion of the toxic factor by ENV1 is linked to iron-dependent metabolism.

**TABLE 2:** Survival of *T. pyriformis* when exposed to CFS from V. vulnificus ENV1 and 0.5× VNSS medium with and without the physical and chemical treatments for 2 h

CFS/medium/treatment	pH^*a*^	*T. pyriformis* survival^*b*^
ENV1 CFS	4.6	Dead
Heat treatment (2 h, −95°C)	4.6	Dead
Freeze/thaw (−20°C)	4.6	Dead
Ultrafiltration (Amicon Ultra-0.5-10,000 NMWL)	4.6	Dead
Protease	4.6	Dead
Proteinase K	4.6	Dead
NaOH	7.0	Alive
CFS of ENV1 grown under iron-deplete conditions	5.1	Alive
0.5× VNSS	7.4	Alive
1 mM HCl	6.0	Alive
2 mM HCl	4.5	Alive
3 mM HCl	3.8	Alive
1 mM acetic acid	5.5	Alive
2 mM acetic acid	4.8	Alive
3 mM acetic acid	4.5	Dead
1 mM acetic acid + HCl	3.9	Dead
2 mM acetic acid + HCl	3.8	Dead

a Values are ±0.05.

b *T. pyriformis* cell suspension was considered dead when less than 10% of the cells were active and alive when more than 90% of the cells were active, compared to the total active cells in the untreated control.

The toxicity of the CFS after physical and chemical treatments was also determined (Table 2). Heating at 95°C for 2 h, freezing at −20°C and thawing, or proteases (pronase E and proteinase K) treatments did not affect the CFS toxicity against *T. pyriformis*. Furthermore, ultrafiltration through 0.5 to 10,000 nominal molecular weight limit (NMWL) filters did not alter CFS toxicity, suggesting that the secreted factor was likely smaller than 10 kDa. Collectively, the results indicate that the toxic factor is not an enzyme or a protein. The CFS pH was approximately 4, and therefore, the role of pH in toxicity was examined. Neutralization of the pH to 7 using sodium hydroxide rendered the CFS nontoxic to *T. pyriformis* (Table 2). Furthermore, to validate if acidity kills *T. pyriformis*, the pH of sterile VNSS medium was adjusted to below 4.6 using hydrochloric acid. This acidified VNSS did not affect the viability of *T. pyriformis*, confirming that the toxicity was not because of the acidity but a toxic factor that is sensitive to pH differences (Table 2).

### V. vulnificus ENV1 kills *T. pyriformis* by excreting acetic acid.

The CFS analysis indicated that the toxic factor is not an enzyme or a protein (heat treatment), and thus, is likely a small molecule (ultrafiltration), and that its function is affected by protonation of functional groups (active in acidic pH). Free fatty acids are small organic compounds produced by several bacteria that have antimicrobial properties, which are dependent on the pH of the environment (30). Therefore, the CFS was tested for the presence of the short-chain fatty acids (SCFAs) acetate, propionate, butyrate, and valerate using gas chromatography. The CFS contained approximately 4 mM acetate, while the other SCFAs could not be detected (Fig. 3C). Addition of acetic acid to sterile VNSS made the medium acidic depending on the concentration of the fatty acid. The medium supplemented with 1 and 2 mM acetic acid changed the pH of the media to 5.5 and 4.8, respectively, but was nontoxic to *T. pyriformis*. However, 3 mM acetic acid resulted in a pH of 3.8 and killed the ciliates (Table 2). Furthermore, adjusting the pH of the medium containing 1 and 2 mM acetic acid to approximately 4 rendered the medium toxic to *T. pyriformis* (Table 2). These results suggest that the toxic factor excreted by the strain ENV1 is acetic acid and that it is active against *T. pyriformis* in its protonated form (acetic acid pKa = 4.6) under acidic pH.

The CFS was toxic to *T. pyriformis* only when ENV1 grew in the presence of iron (Fig. 3A and B), and in the absence iron, the concentration of acetate was significantly lower than that in iron-replete conditions (Fig. 3C and D). The growth of ENV1 without iron was unaffected (Fig. 3E), and change in pH of the iron-depleted CFS was significantly slower (relatively alkaline) than that for the WT (Fig. 3F). The low concentration of acetate in the CFS during iron depletion suggests that acetate excretion of ENV1 is dependent on iron.

### V. vulnificus ENV1 excretes excess acetic acid through overflow metabolism.

To understand the iron-dependent mechanism of acetate excretion in ENV1, we analyzed the transcriptomes of ENV1 cultures grown under iron-replete and iron-depleted conditions by RNA sequencing (Table 3, Table S1).

**TABLE 3:** Differential expression of genes involved in glycolysis, the repression of tricarboxylic acid cycle (TCA), pyruvate fermentation, acetate excretion, and other oxidized metabolites; a complete list of all differentially expressed genes is provided in the supplementary information (Table S1)

Gene locus ID	Expression fold change (log2)	Adjusted *P* value	Gene annotation	Function
Glycolysis, glucose to pyruvate				
BJD94_12875	1.9217	2.125E−08	Glucose-6-phosphate isomerase, *gpi*	Glucose-6-phosphate to fructose-6-phosphate
BJD94_12175	1.8042	2.374E−07	6-Phosphofructokinase, *pfkA*	Fructose 6-phosphate to fructose 1,6-bisphosphate
BJD94_12630	1.379	1.121E−07	Triosephosphate isomerase, *tpiA*	Dihydroxyacetone phosphate (DHAP) to d-glyceraldehyde-3-phosphate (G3P)
BJD94_12290	1.3784	6.156E−10	22C3-bisphosphoglycerate-independent phosphoglycerate mutase, *gpmI*	2-Phosphoglycerate (2-PGA) and 3-phosphoglycerate (3-PGA)
BJD94_13755	1.345	0.0002797	Enolase, *eno*	2-PGA to phosphoenolpyruvate
BJD94_09310	1.2669	0.0011528	Pyruvate kinase, *pyk*	Phosphoenolpyruvate to pyruvate
Pyruvate fermentation to acetyl-CoA				
BJD94_14015	−1.7379	0.34873409	Dihydrolipoamide dehydrogenase of pyruvate dehydrogenase complex, *pdhC*	Aerobic decarboxylation of pyruvate to acetyl-CoA
BJD94_08715	3.8163	3.65E−108	Pyruvate-formate lyase, *tdcE* or *grcA*	Pyruvate to acetyl-CoA and formate
BJD94_01215	4.2489	0	Pyruvate-formate lyase, *pfl*	Pyruvate to acetyl-CoA and formate
Formate dehydrogenation to CO_2_				
BJD94_18845	1.922	0.0009791	NAD-dependent formate dehydrogenase alpha subunit, *fdhA*	Formate to CO_2_
Repression of tricarboxylic acid (TCA) cycle				
BJD94_00805	1.9081	0.0005814	Phosphohistidine phosphatase, *SixA*	Regulation of arcB/arcA two-component system
BJD94_08750	1.8356	2.715E−08	Anaerobic aerobic respiration control protein, arcA	Represses TCA cycle
Acetate metabolism				
BJD94_01780	1.5263	4.2E−06	Acetate kinase, *ackA*	Acetyl-CoA to acetate
BJD94_20925	2.391	0.0314181	Acetate kinase, *ackA*	Acetyl-CoA to acetate
BJD94_06220	1.6943	1.142E−36	Alcohol dehydrogenase 3B acetaldehyde dehydrogenase, *adh/aldh*	Acetyl-CoA to ethanol to acetaldehyde to acetate

Acetate excretion in *Gammaproteobacteria* such as *Vibrio* spp. and Escherichia coli follows two different pathways: (i) direct oxidation of pyruvate by pyruvate oxidase (PoxB) and (ii) decarboxylation of pyruvate to acetyl-CoA, followed by the conversion of acetyl-CoA to acetate by phosphotransacetylase (Pta) and acetate kinase (AckA). Decarboxylation of pyruvate to acetyl-CoA can occur both aerobically, by pyruvate dehydrogenase complex (PdhC), and anaerobically, by pyruvate-formate lyase (Pfl) (31). V. vulnificus strains lack *poxB*, and therefore direct oxidation of pyruvate to acetate is not possible. Under iron-replete conditions, *pdhC* (BJD94_14015) was downregulated and *pfl* genes (BJD94_01215 and BJD94_08715) were upregulated (Table 3, Table S1), suggesting that the pyruvate is converted to acetyl-CoA by fermentation. Furthermore, ENV1 carries two copies of *ackA* genes (BJD94_01780 and BJD94_2925), both of which were upregulated under iron-replete conditions. This indicates that conversion of acetyl-CoA to acetate follows the Pta-AckA pathway and suggests that ENV1 is adapted for acetate excretion through pyruvate fermentation. Moreover, the upregulation of genes associated with alcohol/acetaldehyde dehydrogenase (*adh/aldH*) (BJD94_06220) suggested that acetate accumulates through conversion of acetyl-CoA to ethanol and acetaldehyde and contributes to replenishing NAD^+^ from NADH. In addition to decarboxylation of pyruvate, Pfl also catalyzes pyruvate to formate, which is a metabolic intermediate that also helps replenish the NAD^+^ pool. Furthermore, *fdhA* (BJD94_18845, formate dehydrogenase) that converts formate to carbon dioxide was also upregulated under the iron-replete conditions (Table 3).

**FIG 4:**
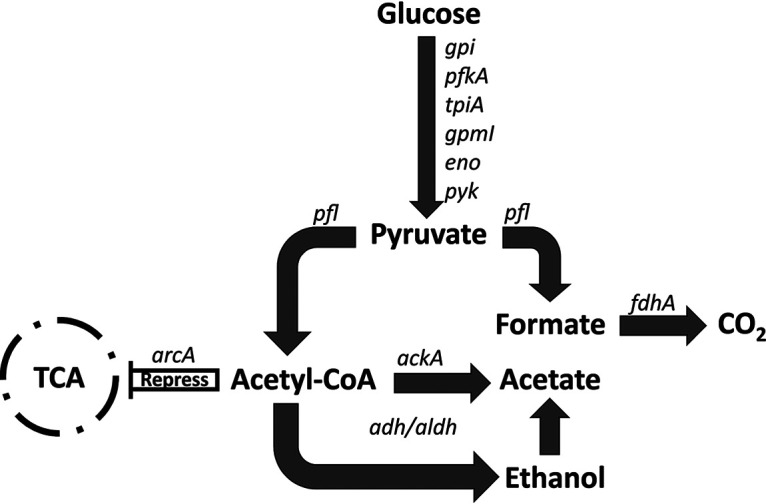
A model based on the genes that are upregulated under iron-replete conditions describing the overflow metabolism that enables synthesis and excretion of acetate by V. vulnificus ENV1, based on the RNA sequence analysis (Table 3). The master regulator, *arcA*, represses the TCA cycle, and as a result the acetyl-CoA is diverted to the *pta-ackA* pathway and leads to excretion of acetate. g*pi*, glucose-6-phosphate; *pfkA*, phosphofructokinase; *tpiA*, triosephosphate isomerase; *gpmI*, phosphoglycerate mutase; *eno*, enolase; *pyk*, pyruvate kinase; *arcA*, anaerobic regulator; *pfl*, pyruvate-formate lyase; *fdhA*, formate dehydrogenase; *ackA*, acetate kinase; aldh, acetaldehyde dehydrogenase; *adh*, alcohol dehydrogenase.

**FIG 5:**
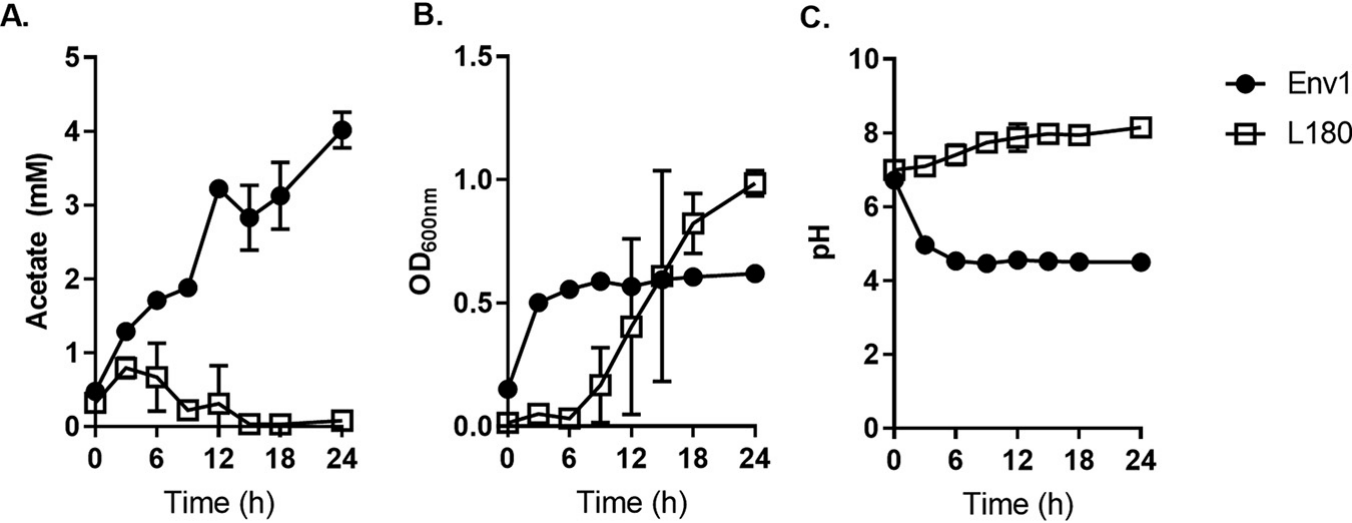
Acetate excretion (A), growth at OD_600_ (B), and pH (C) of V. vulnificus strains ENV1 (filled circles) and the grazing-sensitive L180 (empty squares), under aerobic conditions.

Acetate excretion through the Pta-AckA pathway in E. coli (31), as well as in V. cholerae (32), is associated with the suppression of TCA cycle enzymes by the anaerobic respiration control protein, ArcA (BJD94_08750), which was upregulated under iron-replete conditions (Table 3). Together with the upregulation of *ackA*, *pfl*, *adh*/*aldh*, and *fdhA* and the downregulation of *pdhC*, the transcriptome data suggested that ENV1 generates ATP primarily by excreting acetate (*pfl*, *ackA*) and regenerates NAD^+^ by excreting partially oxidized intermediates, such as ethanol and formate (*adh*, *pfl*), through pyruvate fermentation (Fig. 4). These phenotypes are characteristic of anaerobic growth despite the experiments being conducted under aerobic conditions. Env1 grew at a higher rate during the first 3 h and did not appear to grow further (Fig. 3E, Fig. 5B), which is a characteristic growth pattern for overflow metabolism (31). Several microorganisms use overflow metabolism when carbon flux through glycolysis is higher than that for the tricarboxylic acid (TCA) cycle, resulting in the fermentation of pyruvate to acetate for energy generation instead of respiration, despite the presence of oxygen. Transcriptome analysis indicated that ENV1 was actively expressing genes associated with the glycolysis pathway and had repressed TCA cycle genes. This was based on the upregulation of both *arcA* and fermentation pathway genes involved in conversion of pyruvate to acetate (Fig. 4, Table 3). The above phenotype was confirmed by estimation of acetate in the CFS (Fig. 3C and D, Fig. 5A), which strongly suggested that V. vulnificus ENV1 has adapted to overflow metabolism.

### Acetate excretion is controlled by the anaerobic respiration control protein, ArcA.

To determine if *arcA* controls acetate excretion, and hence predation defense, in ENV1, *arcA* was deleted. The Δ*arcA* mutant was more grazing sensitive than the WT, and the number of predators also increased significantly compared to that of WT ENV1 (Fig. 3A and B). Deletion of *arcA* did not affect the growth of the mutant, but the acetate concentrations in the CFS of Δ*arcA* was significantly lower than that of the WT, suggesting that a lower acetate excretion rate is linked to the grazing sensitivity of the mutant ENV1 (Fig. 3A, D, and E). Therefore, it is clear that *arcA* influences predator defense by controlling the rate of acetate excretion of ENV1.

### Loss of the acetate switch leads to excess acetate accumulation by ENV1.

The acetate switch is a phenomenon when cells switch from acetate excretion through overflow metabolism to assimilation through the Pta-AckA or the PoxB (if present) pathways. For example, a grazing-sensitive V. vulnificus strain (L180) initially excreted acetate but switched to assimilation after 3 h (Fig. 5A). In contrast, ENV1 continued to excrete acetate without subsequent reassimilation over 24 h, suggesting that acetate switch phenomenon is absent in ENV1. Furthermore, L180 had an initial lag phase followed by exponential growth, whereas ENV1 grew faster initially and had no observable lag phase (Fig. 5B). In support of the excess acetate excretion, ENV1 acidified the medium immediately, while L180 showed a slight increase in pH (Fig. 5C). The difference in growth phases and medium acidification indicates a fundamental difference in metabolism. The ability to produce higher concentrations of acetate and the lack of its assimilation, compared to that of L180 (Fig. 5), suggested that ENV1 lacked the acetate switch and has adapted to survive primarily through overflow metabolism.

## DISCUSSION

### Mechanism of predation resistance.

We tested 13 different V. vulnificus strains, representing different genotypes and isolation sources, for resistance to predation by *T. pyriformis*. Only ENV1 showed resistance to predation by *T. pyriformis* (Fig. 1A). Furthermore, coincubation with ENV1 resulted in growth inhibition and toxicity to *T. pyriformis* (Fig. 1B, Fig. 2). This toxicity was also observed for the CFS of ENV1 (Table 2).

### Acetate is secreted and acts as a predation defense for ENV1.

The CFS of ENV1, when subjected to ultrafiltration, protease treatment, or heating and freezing, was toxic to *T. pyriformis*. However, neutralizing the original pH (approximately 4) by adding NaOH rendered the CFS nontoxic, indicating that the active ingredient that confers toxicity is less likely to be a protein or enzyme but a small compound that is sensitive to pH, such as organic acids. High concentrations of acetate (up to 4 mM) were detected in the CFS of ENV1 (Fig. 3C, Fig. 5A), while no other SCFA could be detected (Fig. 3C). Furthermore, when acetate was added to the fresh medium and the pH of the medium was lower than its pKa (4.6), *T. pyriformis* was killed (Table 2).

SCFAs are weak acids and, when the carboxylic group is protonated, can diffuse across cell membranes and dissociate releasing H^+^ ions in the cells. Intracellular protonated SCFAs contribute to increases in hydrogen ion concentrations, resulting in an unfavorable intracellular pH leading to compromised cellular function (33–35). *T. pyriformis* growth is sensitive to changes in intracellular pH. For example, nigericin, an antibiotic derived from *Streptomyces* spp., is a carboxylic polyether compound that acts as an ionophore and an antiporter of H^+^ and K^+^ ions. Treatment with nigericin results in an acidified intracellular environment for *T. pyriformis* (36, 37) and is hence toxic. Additionally, weak acids can perturb cell membranes through disruption of electron transport chains, compromise ATP synthesis by interfering with oxidative phosphorylation, and generate free radicals that cause membrane lysis by forming peroxides or autooxidation products (38, 39). Treatment of *T. pyriformis* with ENV1 CFS or the VNSS medium supplemented with acetic acid led to leakage of the cytoplasm out of the cell membrane (Fig. 2). Collectively, the data suggest that acetate secretion by V. vulnificus ENV1 is the mechanism of grazing resistance against *T. pyriformis*.

V. vulnificus is ferrophilic, and iron plays a key role in pathogenicity (29, 40). The CFS from cells grown under iron-depleted conditions contained significantly less acetate and were no longer toxic to *T. pyriformis* (Fig. 3D, Table 2). In addition, the pH of the culture medium was above the pKa of acetic acid when grown under iron-depleted conditions (Table 2). The change in toxicity, the acetate concentration, and the pH indicate that iron plays a key role in the acetate excretion and the acidification of the environment and, therefore, the defense of ENV1 against *T. pyriformis*.

### Acetate excretion.

Acetogenesis, the excretion of acetate, generates energy and recycles coenzyme A. Acetate excretion occurs either by direct oxidation of pyruvate catalyzed by pyruvate oxidase (PoxB) under aerobic conditions or by decarboxylation of pyruvate (under both aerobic and anaerobic conditions) to acetyl-CoA followed by generation of acetyl-phosphate and then acetate, catalyzed by phosphotransacetylase (Pta) and acetyl kinase (AckA). ENV1, like most *Vibrio* spp., lacks *poxB* and likely decarboxylates pyruvate to acetyl-CoA and converts it to acetate through the Pta-AckA pathway. Decarboxylation of pyruvate to acetyl-CoA can occur both aerobically by pyruvate dehydrogenase complex (PdhC) and anaerobically by pyruvate-formate lyase (Pfl) (31). ENV1 encodes both enzymes and carries *pta* and *ackA* genes in chromosome one and a second copy of *ackA* in chromosome two (41). Transcriptomic analysis of ENV1 grown under iron-replete compared to that grown under iron-depleted conditions showed that *pdhC* was repressed, while *pfl* was induced, suggesting decarboxylation of pyruvate to acetyl-CoA without the need for oxygen. Furthermore, both copies of *ackA* were upregulated in the presence of iron, confirming the involvement of the Pta-AckA pathway in the conversion of acetyl-CoA to acetate (Table 3).

In E. coli, anaerobic respiration is regulated by ArcA that represses TCA cycle genes (42, 43). ArcA is also involved in regulating overflow metabolism, where cells enter a fermentative growth phase despite the presence of oxygen resulting in faster growth but with deceased ATP production per molecule of glucose. TCA cycle enzymes are repressed by ArcA expression, diverting the carbon flux toward the excretion of acetate to generate energy (44–46). Here, *arcA* was upregulated in ENV1 under iron-replete conditions and the transcripts of TCA cycle genes were not upregulated, indicating that the strain carries out overflow metabolism for energy generation (Fig. 4, Table 3). The higher acetate concentration (Fig. 3D, Fig. 5A) in the CFS and the higher growth rate (Fig. 3E and Fig. 5B) also confirmed that ENV1 was undergoing overflow metabolism. The Δ*arcA* mutant produced less acetate (Fig. 3D) and was sensitive to *T. pyriformis* grazing (Fig. 3A), confirming that the acetate excretion was dependent on *arcA*.

While acetate excretion contributes to ATP generation, cells also require NAD^+^ to maintain glycolysis that generates pyruvate from glucose. However, during glycolysis, 2 molecules of NADH are produced for each molecule of glucose oxidized, depleting NAD^+^, which is a substrate for the glycolytic enzyme glyceraldehyde-3-phosphate dehydrogenase. Therefore, cells need to replenish the NAD^+^ pool to maintain the glycolytic flux, and this can be achieved by reoxidation of NADH mediated by the excretion of partially oxidized metabolic intermediates such as lactate, formate, and ethanol in the absence of the TCA cycle. While lactate dehydrogenase (*ldh*), the enzyme responsible for lactate synthesis, was not significantly upregulated, lactate and formate were detected at millimolar concentrations in the CFS of ENV1 (Fig. S1). Furthermore, genes responsible for the synthesis of formate (*pfl*) and ethanol (*adh*) were induced (Table 3). Thus, it is evident that V. vulnificus ENV1 is adapted for oxygen-independent overflow metabolism.

### The missing acetate switch.

Many bacteria excrete acetate when an acetogenic substrate is available and then reassimilate the excreted acetate when the extracellular concentrations of the acetate are high and the substrate low. This phenomenon of switching from excretion to assimilation is called the “acetate switch” (31). When we measured the acetate concentration of ENV1 and the grazing-sensitive strain L180, we found that strain L180 was able to excrete acetate and then switch to assimilation when the extracellular concentration of acetate was around 1 mM and did not excrete more acetate over 24 h of growth (Fig. 5A). In contrast, ENV1 continued to excrete acetate without assimilating it over the 24 h of growth under aerobic conditions. This lack of acetate assimilation shows that ENV1 has adapted mechanisms not to switch to acetate assimilation but continues to produce acetate, which results in an acidic environment. The lack of this acetate switch can happen through at least three different routes, which may also be interconnected: (i) maintaining *arcA* expression and activation, (ii) modulation of the cAMP and NAD^+^ pool required for the transcription and activation of acetyl-CoA synthase (47), and (iii) quorum sensing-mediated switching (47, 48). Further studies are required to understand how ENV1 has acquired this adaptation.

It has been suggested that the evolution of emergent pathogens, as well as their persistence in the environment, is in part due to selection pressures associated with predation and the development of various mechanisms of predation resistance (7–9). Resistance to protozoan grazing mediated by the oxygen-independent overflow metabolism in the absence of acetate switch is a previously unknown mechanism of predation defense. While the implication for such an adaptation to pathogenesis in ENV1 is unknown, anaerobic respiration has been shown to induce virulence factor production in other *Vibrio* spp. (32, 49). For example, a transposon insertion mutation in the primary respiration-linked sodium pump (Na^+^NQR) resulted in hypoxic growth in V. cholerae that led to increased transcription of the virulence gene regulator *toxT* that in turn induces the production of the cholera toxin. Furthermore, *arcA* expression-linked repression of TCA cycle and increased acetate excretion are also associated with increased *toxT* transcription (32). ENV1 does not carry *toxT*, and transcription of no known toxic genes was observed in this study. However, given that overflow acetate metabolism of ENV1 is a natural variation of the central carbon metabolism that exists in the environment, it is likely that V. cholerae, like other potential pathogens, can acquire such metabolic adaptation in its natural habitat. It is not clear if V. vulnificus ENV1, which was isolated from an oyster, has adapted to the unique combination of overflow metabolism and the lack of acetate switch as a result of interactions with protozoan predators. However, the discovery of such a strain from the natural environment presents a compelling case for active environmental surveillance for natural variants that might emerge as potential human pathogens.

### Conclusions.

In conclusion, we found that V. vulnificus ENV1 was resistant to predation by *T. pyriformis*. ENV1 has adapted to overflow metabolism, where it ferments pyruvate and generates energy by excreting acetate under aerobic conditions. ArcA, the anaerobic response regulator, plays a key role in the acetate excretion of ENV1 and therefore is indirectly involved in predator defense. Furthermore, the adaptation involves the loss of the ability to assimilate excreted acetate (the acetate switch), and as a result, the excreted acetate and other organic acids acidify the environment. Therefore, acetate excretion under an acidified environment is a novel bacterial antipredator strategy that provides protection for V. vulnificus ENV1.

## MATERIALS AND METHODS

### Strains and growth conditions.

All V. vulnificus strains were kindly provided by J.D. Oliver, UNC Charlotte, Charlotte, NC and Shin-Ichi Miyoshi of Okayama University, Japan. Bacterial strains (Table 1) were routinely grown in Luria-Bertani broth (LB, Difco, Becton, Dickinson, NJ, USA) supplemented with 2% NaCl and on agar plates (50) as appropriate, with carbenicillin (100 μg mL^−1^). The iron chelator, 2-2′ bipyridyl (BPD) (100 μM) (Sigma-Aldrich, MO, USA), was added to the medium to induce iron limitation. *T. pyriformis* was routinely passaged in 15 mL peptone-yeast-glucose (PYG) medium (20 g L^−1^ proteose peptone, 1 g L^−1^ yeast extract) added to 1 L 0.1× M9 minimal medium (6 g L^−1^ NaH_2_PO_4_, 3 g L^−1^ K_2_PO_4_, 0.5 g L^−1^ NaCl, 1 g L^−1^ NH_4_Cl) supplemented with 0.1 M sterile-filtered glucose in 25 cm^2^ tissue culture flasks with ventilated caps (Sarstedt Inc., Nümbrecht, Germany) and incubated statically at room temperature (RT) for 3 days. To remove the nutrient media and to acclimatize the ciliate to phagotrophic feeding, 500 μL of the *T. pyriformis* culture was added to 20 mL of 0.5× NSS medium (8.8 g L^−1^ NaCl, 0.735 g L^−1^ Na_2_SO_4_, 0.04 g L^−1^ NaHCO_3_, 0.125 g L^−1^ KCl, 0.02 g L^−1^ KBr, 0.935 g L^−1^ MgCl_2_·6 H_2_O, 0.205 g L^−1^ CaCl_2_·2 H_2_O, 0.004 g L^−1^ SrCl_2_·6 H_2_O, and 0.004 g L^−1^ H_3_BO_3_) (51) supplemented with 1% heat-killed Pseudomonas aeruginosa PAO1 in a 25 cm^2^ tissue culture flask and incubated at RT statically for 2 days. The heat-killed bacteria (HKB) were prepared as described previously (18). The health of *T. pyriformis* in each flask was determined by inverted phase contract microscopy. The healthy ciliates are fast swimming and distributed throughout the media. Total numbers of *T. pyriformis* were determined by use of a hemocytometer viewed under bright field light microscopy of three 10 μL aliqouts fixed with 1% Lugol solution (Sigma) (1:1). Videos and images were recorded under these conditions. Uronema marinum (Dujardin 1841) was isolated by Martina Erken in 2011 at the Sydney Institute of Marine Science (SIMS) harbor and kept as a nonaxenic culture.

### Quantification of *T. pyriformis* predation of planktonic cells.

To assess predation of planktonic V. vulnificus, 10^6^ cells mL^−1^ of overnight cultures in 0.5× VNSS (1 g bacteriological peptone, 0.5 g yeast extract, 0.5 g d-glucose, 0.01 g FeSO_4_·7H_2_O, and 0.01 g Na_2_HPO_4_ in 1 L of 0.5× NSS) (52) were added to 24-well microtiter plates (BD Falcon, Becton, Dickinson, NJ, USA). *T. pyriformis* was subsequently added to each well (10^4^ cell mL^−1^; determined by microscopy) and the plates were incubated at RT with shaking at 60 rpm for 24 h. The cell density of each well was measured at an optical desntiy at 600 nm (OD_600_) (Eppendorf PlateReader AF2200, Hamburg, Germany). Planktonic fractions were collected for CFU mL^−1^ counts. *T. pyriformis* was enumerated by microscopy.

### Supernatant toxicity assay.

To determine if factors secreted by V. vulnificus ENV1 were toxic to protozoa, overnight cultures of V. vulnificus ENV1 were adjusted to 10^6^ cells mL^−1^ in 0.5× VNSS and incubated for 24 h. Cell-free supernatants (CFS) were collected by centrifugation at 3,100 × *g* for 5 min and filtered (0.22 μm, Millipore; Bedford, MA, USA). Various treatments of the CFS, including heating (95°C for 2 h), freezing/thawing (–20°C for 24 h), ultrafiltration (Amicon Ultra-0.5-10, 000NMWL), proteinase K (200 μg mL^−1^) from Tritirachium album (Sigma-Aldrich, MO, USA), proteinase (1 mg mL^−1^) from Streptomyces griseus (pronase E) (Sigma-Aldrich, MO, USA), and pH adjustment with hydrochloric acid (HCl, 1 to 3 mM) and sodium hydroxide (NaOH 1 to 3 mM) were tested to assess what types of biomolecules may be responsible for toxicity. The CFS was added to 24-well microtiter plates (BD Falcon, Becton, Dickinson, NJ, USA) containing *T. pyriformis* (10^4^ cell mL^−1^), and numbers of live *T. pyriformis* cells were determined by microscopy. Sterile medium controls (0.5× VNSS) were included for each treatment to ensure none of the treatments were toxic to *T. pyriformis*. Images of the whole field of view at different time points were taken using inverted phase-contrast microscopy. Cells that die due to cytotoxicity lose their shape, become more spherical, and leak cytoplasm through compromised cell membranes. These cells sink to the bottom of the well. Hence, only misshapen, disintegrating cells at the bottom of the well were counted as dead.

### Estimation of fatty acid content.

For the estimation of short-chain fatty acids (acetate, propionate, butyrate, and valerate), CFS was collected as described previously. CFS was acidified with formic acid (0.1% final concentration) and analyzed by gas chromatography flame ionization detector (GC-FID) using a DB-FFAP column (Agilent) with standards of each fatty acid prepared in water at concentrations between 1 and 200 ppm.

### RNA extraction and sequencing.

Overnight cultures of V. vulnificus were adjusted to 10^6^ cells mL^−1^ (OD_600_ = 0.001) in 0.5× VNSS (iron-replete) or 0.5× VNSS supplemented with a 100 μM concentration of the iron chelator, 2-2′ dipyridyl (iron-depleted) in 24-well microtiter plates (BD Falcon, Becton, Dickinson, NJ, USA). Plates were incubated for 10 h at RT with shaking at 60 rpm (early stationary phase), and the supernatant toxicity was determined. The samples were fixed in RNAprotect bacteria reagent (Qiagen). Total RNA was extracted by lysozyme digestion and the RNeasy plus minikit (Qiagen) following the manufacturer’s instructions. RNA concentration and purity were determined by spectrophotometer (NanoDrop ND-1000), and the integrity of the RNA was determined by agarose gel electrophoresis and using an Agilent Bioanalyzer 2100. The RNA was stored at −80°C until it was prepared for sequencing using the Illumina standard kit following the manufacturer’s protocol (Illumina). Samples were sequenced by paired-end sequencing on the Illumina Hi-Seq 2500 platform with read lengths of 100 bp.

### Transcriptome data analysis.

The quality of the paired-end reads was initially checked using FastQC (https://www.bioinformatics.babraham.ac.uk/projects/fastqc/). Illumina adaptors, short reads, and low-quality reads were removed using cutadapt (version 1.11) (53). *In silico* rRNA depletion for high-quality reads (97% to 98% of the raw reads) was performed using sortmeRNA (version 2.0) (54). mRNA reads (from 75,495 to 131,958 read pairs) were then mapped to the V. vulnificus ENV1 genome (41) using Bowtie2 (version 2.2.9) (55). The number of reads mapping to each gene was determined using HTSeq (version v.0.6.1p1) (56).

The raw count table of transcripts was then used as an input for the Deseq2 R package for differential expression analysis (57). Briefly, the raw counts were normalized according to sample library size and a negative binomial test was performed to identify the differentially expressed genes. Genes were considered differentially expressed if their absolute fold change value was greater than 2 and the associated adjusted *P* value was smaller than 0.05. The normalized transcripts were then log_2_ (N + 1) transformed prior to principal-component analysis and unweighted pair group method using average linkages (UPGMA) hierarchical clustering for the sample dendrogram on the heatmap.

### Generation of *arcA* null mutation.

A four-fragment construct was generated using NEBuilder HiFi DNA assembly master mix (New England Biolabs), consisting of a 750-bp region upstream of the *arcA* start codon, a 750-bp region downstream of *arcA*, a gentamicin resistance cassette, and the linearized suicide vector pCVD442 (Addgene no. 11074). The assembled construct was transformed into E. coli BW20767 via heat shock transformation. The correct insertion of fragments in pCVD442 was confirmed by sequencing (Sanger sequencing, 1st Base, Singapore).

The construct was introduced into V. vulnificus ENV1 using electroporation (58). Electrocompetent cells were produced by washing mid-log-phase ENV1 cells with 400 mM sucrose at RT. After electroporation (10 kV/cm, 25 μF, 200 Ω), cells were recovered in Super Optimal broth with catabolite repression (SOC) supplemented with 2% NaCl for 3 h at 37°C. The *arcA* mutant cells were selected using ABTC medium supplemented with 60 μg mL^−1^ gentamicin at 30°C for 96 h. ABTC medium consists of solutions A and B of the defined growth medium described by Clark and Maaløe (59) supplemented with 2.5 mg L^−1^ thiamine (T) and 10 mM citrate (C). Counter selection of the clones was achieved by spreading mid-log-phase cultures of the 1st recombination clones on counter selection agar containing sucrose (10 g L^−1^ peptone, 5 g L^−1^ yeast extract, 2 g L^−1^ sodium chloride, gentamicin 60 μg mL^−1^, and 15% vol/vol sucrose). The deletion was confirmed by PCR and sequencing of the PCR product (Sanger sequencing, 1st Base, Asia).

### Data analysis.

Statistical analyses were performed using GraphPad Prism version 7.03 for Windows (GraphPad Software, La Jolla, CA, USA). Data that did not follow Gaussian distribution, as determined by frequency distribution graphs, were natural log transformed. Two-tailed student’s *t* tests were used to compare means between experimental samples and controls. For experiments including multiple samples, one-way or 2-way analyses of variance (ANOVAs) were used for the analysis and Sidak’s or Dunnett’s multiple-comparison test provided *post hoc* comparison of means when appropriate.

### Data availability.

Data from this study have been deposited in the NCBI database under BioProject accession number PRJNA448800, BioSample accession number SAMN08866536, and SRA accession numbers SRR6942709, SRR6942712, SRR6942711, SRR6942710, SRR6942713, and SRR6942708.

## References

[1] Oliver JD. 2013. *Vibrio vulnificus*: death on the half shell. A personal journey with the pathogen and its ecology. Microb Ecol 65:793–799. 10.1007/s00248-012-0140-9. 23263234

[2] Horseman MA, Surani S. 2011. A comprehensive review of *Vibrio vulnificus:* an important cause of severe sepsis and skin and soft-tissue infection. Int J Infect Dis 15:e157-66. 10.1016/j.ijid.2010.11.003. 21177133

[3] Rosche TM, Yano Y, Oliver JD. 2005. A rapid and simple PCR analysis indicates there are two subgroups of *Vibrio vulnificus* which correlate with clinical or environmental isolation. Microbiol Immunol 49:381–389. 10.1111/j.1348-0421.2005.tb03731.x. 15840964

[4] Warner EB, Oliver JD. 2008. Multiplex PCR assay for detection and simultaneous differentiation of genotypes of *Vibrio vulnificus* biotype 1. Foodborne Pathog Dis 5:691–693. 10.1089/fpd.2008.0120. 18687035

[5] López-Pérez M, Jayakumar JM, Haro-Moreno JM, Zaragoza-Solas A, Reddi G, Rodriguez-Valera F, Shapiro OH, Alam M, Almagro-Moreno S, Laub MT, Jensen P, Gómez-Consarnau L. 2019. Evolutionary model of cluster divergence of the emergent marine pathogen *Vibrio vulnificus*: from genotype to ecotype. mBio 10:e02852-18. 10.1128/mBio.02852-18. 30782660 PMC6381281

[6] Roig FJ, González-Candelas F, Sanjuán E, Fouz B, Feil EJ, Llorens C, Baker-Austin C, Oliver JD, Danin-Poleg Y, Gibas CJ, Kashi Y, Gulig PA, Morrison SS, Amaro C. 2018. Phylogeny of Vibrio vulnificus from the analysis of the core-genome: implications for intra-species taxonomy. Front Microbiol 8:2613. 10.3389/fmicb.2017.02613. 29358930 PMC5765525

[7] Sun S, Noorian P, McDougald D. 2018. Dual role of mechanisms involved in resistance to predation by protozoa and virulence to humans. Front Microbiol 9:1017. 10.3389/fmicb.2018.01017. 29867902 PMC5967200

[8] Espinoza-Vergara G, Hoque MM, McDougald D, Noorian P. 2020. The impact of protozoan predation on the pathogenicity of *Vibrio cholerae*. Front Microbiol 11:17. 10.3389/fmicb.2020.00017. 32038597 PMC6985070

[9] Jones MK, Oliver JD. 2009. *Vibrio vulnificus*: disease and pathogenesis. Infect Immun 77:1723–1733. 10.1128/IAI.01046-08. 19255188 PMC2681776

[10] Matz C, Kjelleberg S. 2005. Off the hook–how bacteria survive protozoan grazing. Trends Microbiol 13:302–307. 10.1016/j.tim.2005.05.009. 15935676

[11] Hahn MW, Höfle MG. 1998. Grazing pressure by a bacterivorous flagellate reverses the relative abundance of *Comamonas acidovorans*PX54 and *Vibrio* Strain CB5 in chemostat cocultures. Appl Environ Microbiol 64:1910–1918. 10.1128/AEM.64.5.1910-1918.1998. 9572971 PMC106250

[12] Hahn MW, Moore ER, Höfle MG. 1999. Bacterial filament formation, a defense mechanism against flagellate grazing, is growth rate controlled in bacteria of different phyla. Appl Environ Microbiol 65:25–35. 10.1128/AEM.65.1.25-35.1999. 9872755 PMC90978

[13] Wildschutte H, Wolfe DM, Tamewitz A, Lawrence JG. 2004. Protozoan predation, diversifying selection, and the evolution of antigenic diversity in *Salmonella*. Proc Natl Acad Sci USA 101:10644–10649. 10.1073/pnas.0404028101. 15247413 PMC489988

[14] Matz C, McDougald D, Moreno AM, Yung PY, Yildiz FH, Kjelleberg S. 2005. Biofilm formation and phenotypic variation enhance predation-driven persistence of *Vibrio cholerae*. Proc Natl Acad Sci USA 102:16819–16824. 10.1073/pnas.0505350102. 16267135 PMC1283802

[15] Jousset A. 2012. Ecological and evolutive implications of bacterial defences against predators. Environ Microbiol 14:1830–1843. 10.1111/j.1462-2920.2011.02627.x. 22040156

[16] Chavez-Dozal A, Gorman C, Erken M, Steinberg PD, McDougald D, Nishiguchi MK. 2013. Predation response of *Vibrio fischeri* biofilms to bacterivorus protists. Appl Environ Microbiol 79:553–558. 10.1128/AEM.02710-12. 23144127 PMC3553747

[17] Matz C, Deines P, Boenigk J, Arndt H, Eberl L, Kjelleberg S, Jurgens K. 2004. Impact of violacein-producing bacteria on survival and feeding of bacterivorous nanoflagellates. Appl Environ Microbiol 70:1593–1599. 10.1128/AEM.70.3.1593-1599.2004. 15006783 PMC368400

[18] Noorian P, Hu J, Chen Z, Kjelleberg S, Wilkins MR, Sun S, McDougald D. 2017. Pyomelanin produced by *Vibrio cholerae* confers resistance to predation by *Acanthamoeba castellanii*. FEMS Microbiol Ecol 93. 10.1093/femsec/fix147. PMC5812506 29095994

[19] Sun S, Tay QX, Kjelleberg S, Rice SA, McDougald D. 2015. Quorum sensing-regulated chitin metabolism provides grazing resistance to *Vibrio cholerae* biofilms. ISME J 9:1812–1820. 10.1038/ismej.2014.265. 25615438 PMC4511936

[20] Lainhart W, Stolfa G, Koudelka GB. 2009. Shiga toxin as a bacterial defense against a eukaryotic predator, *Tetrahymena thermophila*. J Bacteriol 191:5116–5122. 10.1128/JB.00508-09. 19502393 PMC2725575

[21] Pukatzki S, Ma AT, Sturtevant D, Krastins B, Sarracino D, Nelson WC, Heidelberg JF, Mekalanos J. 2006. Identification of a conserved bacterial protein secretion system in *Vibrio cholerae* using the *Dictyostelium* host model system. Proc Natl Acad Sci USA 103:1528–1533. 10.1073/pnas.0510322103. 16432199 PMC1345711

[22] Pukatzki S, Ma AT, Revel AT, Sturtevant D, Mekalanos J. 2007. Type VI secretion system translocates a phage tail spike-like protein into target cells where it cross-links actin. Proc Natl Acad Sci USA 104:15508–15513. 10.1073/pnas.0706532104. 17873062 PMC2000545

[23] Miyata ST, Kitaoka M, Brooks TM, McAuley SB, Pukatzki S. 2011. *Vibrio cholerae* requires the Type VI SecretionSystem virulence factor VasX to kill *Dictyostelium discoideum*. Infect Immun 79:2941–2949. 10.1128/IAI.01266-10. 21555399 PMC3191968

[24] Vaitkevicius K, Lindmark B, Ou G, Song T, Toma C, Iwanaga M, Zhu J, Andersson A, Hammarström M-L, Tuck S, Wai SN. 2006. A *Vibrio cholerae* protease needed for killing of *Caenorhabditis elegans* has a role in protection from natural predator grazing. Proc Natl Acad Sci USA 103:9280–9285. 10.1073/pnas.0601754103. 16754867 PMC1482601

[25] Sun S, Kjelleberg S, McDougald D. 2013. Relative contributions of Vibrio polysaccharide and quorum sensing to the resistance of *Vibrio cholerae* to predation by heterotrophic protists. PLoS One 8:e56338. 10.1371/journal.pone.0056338. 23441178 PMC3575383

[26] Erken M, Weitere M, Kjelleberg S, McDougald D. 2011. In situ grazing resistance of *Vibrio cholerae* in the marine environment. FEMS Microbiol Ecol 76:504–512. 10.1111/j.1574-6941.2011.01067.x. 21314704

[27] Lee CT, Pajuelo D, Llorens A, Chen YH, Leiro JM, Padros F, Hor LI, Amaro C. 2013. MARTX of *Vibrio vulnificus* biotype 2 is a virulence and survival factor. Environ Microbiol 15:419–432. 10.1111/j.1462-2920.2012.02854.x. 22943291

[28] Kim BS. 2018. The modes of action of MARTX toxin effector domains. Toxins (Basel) 10:507. 10.3390/toxins10120507. PMC6315884 30513802

[29] Wright AC, Simpson LM, Oliver JD. 1981. Role of iron in the pathogenesis of *Vibrio vulnificus* infections. Infect Immun 34:503–507. 10.1128/iai.34.2.503-507.1981. 7309236 PMC350895

[30] Yoon BK, Jackman JA, Valle-Gonzalez ER, Cho NJ. 2018. Antibacterial free fatty acids and monoglycerides: biological activities, experimental testing, and therapeutic applications. Int J Mol Sci 19:1114. 10.3390/ijms19041114. PMC5979495 29642500

[31] Wolfe AJ. 2005. The acetate switch. Microbiol Mol Biol Rev 69:12–50. 10.1128/MMBR.69.1.12-50.2005. 15755952 PMC1082793

[32] Minato Y, Fassio SR, Wolfe AJ, Hase CC. 2013. Central metabolism controls transcription of a virulence gene regulator in *Vibrio cholerae*. Microbiology (Reading) 159:792–802. 10.1099/mic.0.064865-0. 23429745 PMC3709826

[33] Ullah A, Orij R, Brul S, Smits GJ. 2012. Quantitative analysis of the modes of growth inhibition by weak organic acids in *Saccharomyces cerevisiae*. Appl Environ Microbiol 78:8377–8387. 10.1128/AEM.02126-12. 23001666 PMC3497387

[34] Jarboe LR, Royce LA, Liu P. 2013. Understanding biocatalyst inhibition by carboxylic acids. Front Microbiol 4:272. 10.3389/fmicb.2013.00272. 24027566 PMC3760142

[35] Gabba M, Frallicciardi J, van 't Klooster J, Henderson R, Syga L, Mans R, van Maris AJA, Poolman B. 2020. Weak acid permeation in synthetic lipid vesicles and across the yeast plasma membrane. Biophys J 118:422–434. 10.1016/j.bpj.2019.11.3384. 31843263 PMC6976801

[36] Bamdad M, Groliere CA, Dupy-Blanc J, Cuer A, David L, Tabet JC. 1993. Microbial conversion of lonophorous antibiotic nigericin by the ciliate *Tetrahymena pyriformis*. Eur J Protistol 29:407–415. 10.1016/S0932-4739(11)80403-7. 23195739

[37] Bamdad M, David L, Groliere CA. 1995. Epinigericin toxicity towards *Tetrahymena pyriformis* GL; changes in cell volume and intracellular pH. Appl Microbiol Biotechnol 44:206–209. 10.1007/BF00164503. 8579832

[38] Ricke SC. 2003. Perspectives on the use of organic acids and short chain fatty acids as antimicrobials. Poult Sci 82:632–639. 10.1093/ps/82.4.632. 12710485

[39] Desbois AP, Smith VJ. 2010. Antibacterial free fatty acids: activities, mechanisms of action and biotechnological potential. Appl Microbiol Biotechnol 85:1629–1642. 10.1007/s00253-009-2355-3. 19956944

[40] Kim CM, Park RY, Choi MH, Sun HY, Shin SH. 2007. Ferrophilic characteristics of *Vibrio vulnificus* and potential usefulness of iron chelation therapy. J Infect Dis 195:90–98. 10.1086/509822. 17152012

[41] Noorian P, Sun S, McDougald D. 2018. Complete genome sequence of oyster isolate *Vibrio vulnificus* Env1. Genome Announc 6:e00421-18. 10.1128/genomeA.00421-18. 29773632 PMC5958264

[42] Gunsalus RP, Park SJ. 1994. Aerobic-anaerobic gene regulation in *Escherichia coli*: control by the ArcAB and Fnr regulons. Res Microbiol 145:437–450. 10.1016/0923-2508(94)90092-2. 7855430

[43] Chang DE, Smalley DJ, Conway T. 2002. Gene expression profiling of *Escherichia coli* growth transitions: an expanded stringent response model. Mol Microbiol 45:289–306. 10.1046/j.1365-2958.2002.03001.x. 12123445

[44] Vemuri GN, Altman E, Sangurdekar DP, Khodursky AB, Eiteman MA. 2006. Overflow metabolism in *Escherichia coli* during steady-state growth: transcriptional regulation and effect of the redox ratio. Appl Environ Microbiol 72:3653–3661. 10.1128/AEM.72.5.3653-3661.2006. 16672514 PMC1472329

[45] Basan M, Hui S, Williamson JR. 2017. ArcA overexpression induces fermentation and results in enhanced growth rates of *E. coli*. Sci Rep 7:11866. 10.1038/s41598-017-12144-6. 28928483 PMC5605494

[46] Szenk M, Dill KA, de Graff AMR. 2017. Why do fast-growing bacteria enter overflow metabolism? Testing the membrane real estate hypothesis. Cell Syst 5:95–104. 10.1016/j.cels.2017.06.005. 28755958

[47] Wolfe AJ. 2008. Quorum sensing “flips” the acetate switch. J Bacteriol 190:5735–5737. 10.1128/JB.00825-08. 18586946 PMC2519541

[48] Studer SV, Mandel MJ, Ruby EG. 2008. AinS quorum sensing regulates the *Vibrio fischeri* acetate switch. J Bacteriol 190:5915–5923. 10.1128/JB.00148-08. 18487321 PMC2519518

[49] Bueno E, Pinedo V, Cava F. 2020. Adaptation of *Vibrio cholerae* to hypoxic environments. Front Microbiol 11:739. 10.3389/fmicb.2020.00739. 32425907 PMC7212424

[50] Sambrook J, Fritsch EF, Maniatis T. 1989. Molecular cloning: a laboratory manual. Cold Spring Harbor Laboratory Press, New York, NY.

[51] Mårdén P, Tunlid A, Malmcrona-Friberg K, Odham G, Kjelleberg S. 1985. Physiological and morphological changes during short term starvation of marine bacterial islates. Arch Microbiol 142:326–332. 10.1007/BF00491898.

[52] Väätänen P. 1977. Effects of composition of substrate and inoculation technique on plate counts of bacteria in the Northern Baltic Sea. J Appl Bacteriol 42:437–443. 10.1111/j.1365-2672.1977.tb00713.x.

[53] Chen C, Khaleel SS, Huang H, Wu CH. 2014. Software for pre-processing Illumina next-generation sequencing short read sequences. Source Code Biol Med 9:8. 10.1186/1751-0473-9-8. 24955109 PMC4064128

[54] Kopylova E, Noé L, Da Silva C, Berthelot J-F, Alberti A, Aury J-M, Touzet H. 2015. Deciphering metatranscriptomic data, p 279–291, RNA Bioinformatics. Springer. 10.1007/978-1-4939-2291-8_17 25577385

[55] Langmead B, Salzberg SL. 2012. Fast gapped-read alignment with Bowtie 2. Nat Methods 9:357–359. 10.1038/nmeth.1923. 22388286 PMC3322381

[56] Anders S, Pyl PT, Huber W. 2015. HTSeq—a Python framework to work with high-throughput sequencing data. Bioinformatics 31:166–169. 10.1093/bioinformatics/btu638. 25260700 PMC4287950

[57] Love MI, Huber W, Anders S. 2014. Moderated estimation of fold change and dispersion for RNA-seq data with DESeq2. Genome Biol 15:550. 10.1186/s13059-014-0550-8. 25516281 PMC4302049

[58] Jayakumar JM, Shapiro OH, Almagro-Moreno S. 2020. Improved method for transformation of Vibrio vulnificus by electroporation. Curr Protoc Microbiol 58:e106. 10.1002/cpmc.106. 32614522

[59] Clark DJ, Maaløe O. 1967. DNA replication and the division cycle in *Escherichia coli*. J Mol Biol 23:99–112. 10.1016/S0022-2836(67)80070-6.

[60] Kim YR, Lee SE, Kim CM, Kim SY, Shin EK, Shin DH, Chung SS, Choy HE, Progulske-Fox A, Hillman JD, Handfield M, Rhee JH. 2003. Characterization and pathogenic significance of *Vibrio vulnificus* antigens preferentially expressed in septicemic patients. Infect Immun 71:5461–5471. 10.1128/IAI.71.10.5461-5471.2003. 14500463 PMC201039

[61] Poole MD, Oliver JD. 1978. Experimental pathogenicity and mortality in ligated ileal loop studies of the newly reported halophilic lactose-positive *Vibrio* sp. Infect Immun 20:126–129. 10.1128/iai.20.1.126-129.1978. 669787 PMC421561

[62] Hayat U, Reddy GP, Bush CA, Johnson JA, Wright AC, Morris JG, Jr. 1993. Capsular types of *Vibrio vulnificus*: an analysis of strains from clinical and environmental sources. J Infect Dis 168:758–762. 10.1093/infdis/168.3.758. 8354917

[63] Hor LI, Chang YK, Chang CC, Lei HY, Ou JT. 2000. Mechanism of high susceptibility of iron-overloaded mouse to *Vibrio vulnificus* infection. Microbiol Immunol 44:871–878. 10.1111/j.1348-0421.2000.tb02577.x. 11145266

[64] Miyoshi N, Shimizu C, Miyoshi S, Shinoda S. 1987. Purification and characterization of *Vibrio vulnificus* protease. Microbiol Immunol 31:13–25. 10.1111/j.1348-0421.1987.tb03064.x. 3295490

