# Supplementary material for: Loss of the Acetate Switch in Vibrio vulnificus Enhances Predation Defense against Tetrahymena pyriformis
Source: Appl Environ Microbiol. 2022 Jan 25;88(2):e01665-21. doi: 10.1128/AEM.01665-21 (PMC8788688; doi:10.1128/AEM.01665-21)
Supplement: Supplemental file 1 — Methods, Fig. S1, Tables S1 and S2. Download AEM.01665-21-s0001.pdf, PDF file, 0.4 MB [file aem.01665-21-s0001.pdf]

Supplementary material

Methods

Estimation of formate and lactate

The CFS of Env1 at different time points were harvested as described in the methods section of the paper and stored at $4^{o}C$ until analysis. Formate and lactate concentrations were estimated using high performance liquid chromatography (HPLC) with 5 mM sulphuric acid as the mobile phase, using a PDA detector. Standards for formate and lactate were prepared in water at a range of 5 mM to 100 mM.

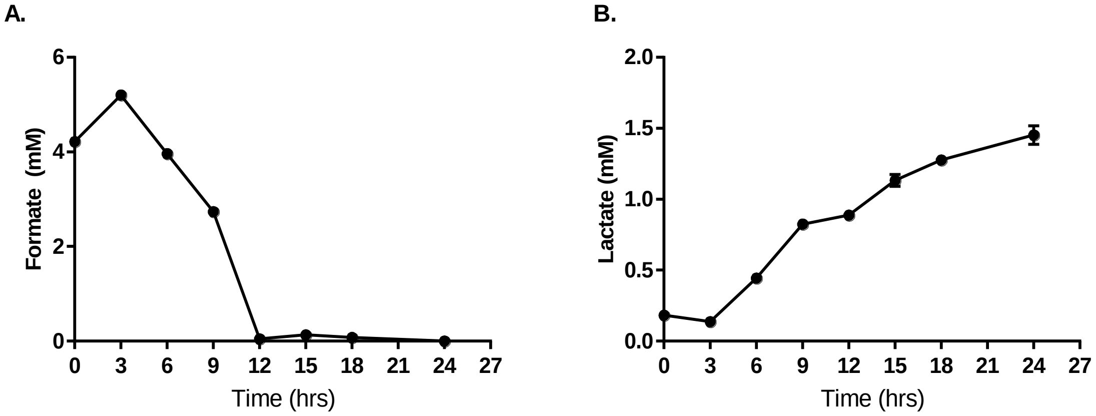

Figure S1. Concentration of formate (mM) and lactate (mM) in the CFS of *V. vulnificus* Env1 wild type when grown under aerobic conditions for up to 24 hours.

Table S1. List of differentially expressed genes

Locus ID	Regulation	Log2 Fold Change	p-adj	Annotation
BJD94_16620	Down	-10.1067	1.429E-15	Non-ribosomal peptide synthetase/ siderophore biosynthesis
BJD94_05245	Down	-9.5466	8.045E-14	Predicted manganese transporter
BJD94_16430	Down	-8.9943	3.62E-12	ABC transporter/ ATP-binding protein
BJD94_08055	Down	-8.7055	1.506E-11	Putative hemolysin
BJD94_05555	Down	-8.4725	1.131E-10	Hypothetical protein
BJD94_16425	Down	-8.3031	3.245E-10	Hypothetical protein
BJD94_12150	Down	-7.9837	1.341E-09	Manganese superoxide dismutase
BJD94_16640	Down	-7.7794	8.542E-09	22C3-dihydroxybenzoate-AMP ligase
BJD94_08065	Down	-7.5952	2.202E-08	Hypothetical protein
BJD94_16670	Down	-7.47	1.95E-08	Catechol siderophore ABC transporter
BJD94_16660	Down	-7.4664	5.297E-08	22C3-dihydroxybenzoate-AMP ligase

BJD94_16655	Down	-7.311	4.489E-08	Isochorismate pyruvate-lyase of siderophore biosynthesis
BJD94_08075	Down	-7.2332	2.137E-10	D-alanine-D-alanine ligase
BJD94_01370	Down	-7.1921	2.323E-07	COG0398: uncharacterized membrane protein
BJD94_16665	Down	-7.1501	2.857E-07	Aryl carrier domain
BJD94_10780	Down	-7.1271	3.302E-07	Putative signal peptide protein
BJD94_08060	Down	-7.0649	7.75E-10	Hypothetical protein
BJD94_16650	Down	-6.9406	3.153E-07	Isochorismatase of siderophore biosynthesis
BJD94_10785	Down	-6.8989	5.665E-07	Uncharacterized protein conserved in bacteria
BJD94_21785	Down	-6.8693	1.395E-06	TonB system biopolymer transport component
BJD94_08070	Down	-6.8437	5.414E-07	Hypothetical protein
BJD94_20305	Down	-6.8229	1.984E-06	Arginine/ornithine antiporter ArcD
BJD94_19940	Down	-6.8221	3.154E-09	Putative heme iron utilisation protein

BJD94_16625	Down	-6.7275	1.013E-06	2-keto-3-deoxy-D-arabino-heptulosonate-7-phosphate synthase I alpha
BJD94_11755	Down	-6.7161	1.027E-06	O-methyltransferase-related protein
BJD94_16645	Down	-6.6692	4.152E-06	Vulnibactin utilisation protein VuuB
BJD94_16630	Down	-6.6636	4.175E-06	22C3-dihydro-22C3-dihydroxybenzoate dehydrogenase
BJD94_19410	Down	-6.6546	1.488E-06	Arginine/ornithine antiporter ArcD
BJD94_16635	Down	-6.6439	1.371E-08	Isochorismate synthase of siderophore biosynthesis
BJD94_19920	Down	-6.5268	9.476E-06	Biopolymer transport protein ExbD1
BJD94_10790	Down	-6.2676	3.382E-05	Glutamate synthase [NADPH] large chain
BJD94_05720	Down	-6.2517	9.94E-116	Peptide methionine sulfoxide reductase MsrA/Peptide methionine sulfoxide reductase MsrB

BJD94_02315	Down	-6.1505	1.593E-05	Hypothetical protein
BJD94_19935	Down	-6.1427	1.121E-24	
BJD94_05550	Down	-6.1059	6.883E-05	Ferric siderophore transport system2C periplasmic binding protein TonB
BJD94_02310	Down	-6.0162	0.0001136	Hypothetical protein
BJD94_16675	Down	-5.923	2.973E-19	Ferric vulnibactin receptor VuuA
BJD94_01845	Down	-5.8087	1.488E-06	Iron-regulated protein A precursor
BJD94_05305	Down	-5.7917	0.000354	Hypothetical protein
BJD94_16615	Down	-5.7728	0.0001022	Non-ribosomal peptide synthetase modules2C siderophore biosynthesis
BJD94_19625	Down	-5.5547	0.0008587	TonB-dependent heme receptor HutR
BJD94_19635	Down	-5.553	0.0009886	Hypothetical protein in cluster with HutR2C VCA0066 homolog
BJD94_01365	Down	-5.514	0.0010025	Hypothetical protein
BJD94_21510	Down	-5.4895	6.368E-21	TonB-dependent heme and hemoglobin receptor HutA %3B

				TonB-dependent hemin 2C ferrichrome receptor
BJD94_21765	Down	-5.4732	0.001202	Ferric siderophore transport system2C periplasmic binding protein TonB
BJD94_17985	Down	-5.4654	0.0013187	Peptide ABC transporter2C ATP-binding protein
BJD94_19260	Down	-5.4644	0.0013816	Hypothetical protein
BJD94_15840	Down	-5.4311	5.844E-30	Glutaredoxin
BJD94_02330	Down	-5.3859	0.0016065	Heat shock protein 22.5
BJD94_19620	Down	-5.3703	0.0005607	Protease II
BJD94_19915	Down	-5.3504	0.0005485	Periplasmic hemin-binding protein
BJD94_17460	Down	-5.2842	0.0006877	Ferric aerobactin ABC transporter2C ATPase component
BJD94_19925	Down	-5.2804	0.0008581	Ferric siderophore transport system2C biopolymer transport protein ExbB
BJD94_05100	Down	-5.278	0.0024007	ABC transporter2C permease protein YnjC

BJD94_19630	Down	-5.2121	0.0036165	Hypothetical with regulatory P domain of a subtilisin-like proprotein convertase
BJD94_20775	Down	-5.2041	2.855E-07	Hypothetical protein
BJD94_19435	Down	-5.1804	0.0050982	Hypothetical protein
BJD94_19945	Down	-5.1493	6.046E-18	Pyridoxamine 5'-phosphate oxidase-related putative heme iron utilisation protein
BJD94_02350	Down	-5.1419	0.0042818	Flp pilus assembly protein TadA
BJD94_17465	Down	-5.0453	0.0067597	Ferric aerobactin ABC transporter2C periplasmic substrate binding protein
BJD94_08080	Down	-5.0142	0.0001236	Hypothetical protein
BJD94_18740	Down	-4.9985	0.0022105	Transcriptional regulator2C AraC family
BJD94_12255	Down	-4.9427	0.0085998	Hypothetical protein
BJD94_15770	Down	-4.9356	2.71E-114	Phosphomannomutase
BJD94_00785	Down	-4.9137	1.308E-33	RNA polymerase sigma-70 factor2C ECF subfamily
BJD94_21780	Down	-4.9127	0.0002191	MotA/TolQ/ExbB proton channel family protein

BJD94_19640	Down	-4.9068	0.0096334	Hypothetical protein in cluster with HutR2C VCA0067 homolog
BJD94_10795	Down	-4.8959	0.0128033	INTEGRAL MEMBRANE PROTEIN (Rhomboid family)
BJD94_10540	Down	-4.8918	9.2E-86	Glutamine synthetase type I glnA
BJD94_16680	Down	-4.7064	0.0005739	Amide synthase component of siderophore synthetase
BJD94_09380	Down	-4.4658	1.403E-06	Uncharacterized protein DUF547
BJD94_00780	Down	-4.4589	2.813E-27	Hypothetical protein
BJD94_14135	Down	-4.4071	5.773E-21	Ferric iron ABC transporter2C iron-binding protein
BJD94_06175	Down	-4.368	0.0022438	Hypothetical protein
BJD94_17865	Down	-4.3277	0.0174521	Hypothetical protein
BJD94_05095	Down	-4.204	2.038E-09	ABC transporter2C periplasmic substrate-binding protein YnjB
BJD94_20430	Down	-4.1459	0.0305584	Signal recognition particle GTPase
BJD94_06915	Down	-3.9229	0.0010037	Ferrous iron transport protein C
BJD94_21320	Down	-3.6724	0.0228542	Hypothetical protein
BJD94_18735	Down	-3.6262	0.0037744	Ferrichrome-iron receptor

BJD94_21760	Down	-3.5251	0.0087474	TPR domain protein2C putative component of TonB system
BJD94_02335	Down	-3.3449	0.0124687	Flp pilus assembly protein2C secretin CpaC
BJD94_17605	Down	-3.2568	1.867E-11	Peptide methionine sulfoxide reductase MsrB
BJD94_04470	Down	-3.154	1.819E-08	Type I secretion system2C outer membrane component LapE
BJD94_05345	Down	-3.043	0.01088	Predicted signal transduction protein
BJD94_15350	Down	-3.0391	1.052E-06	RTX toxins determinant A and related Ca2+-binding proteins rtxA
BJD94_04465	Down	-3.0026	8.671E-37	T1SS secreted agglutinin RTX
BJD94_18620	Down	-2.9442	0.0059387	Hypothetical protein
BJD94_17610	Down	-2.927	4.097E-09	Peptide methionine sulfoxide reductase MsrA
BJD94_11510	Down	-2.8592	4.955E-08	Hypothetical protein
BJD94_07065	Down	-2.5698	6.417E-13	
BJD94_19265	Down	-2.5684	1.904E-21	Uncharacterized conserved protein

BJD94_15725	Down	-2.3333	0.0093729	Outer membrane protein A precursor
BJD94_15005	Down	-2.3094	0.0080751	Methyl-accepting chemotaxis protein
BJD94_12615	Down	-2.3039	2.491E-07	Bacterioferritin-associated ferredoxin
BJD94_19280	Down	-2.3026	0.0024904	Chemotactic transducer-related protein
BJD94_06920	Down	-2.2854	5.288E-23	Ferrous iron transport protein B
BJD94_04475	Down	-2.2767	0.0203348	T1SS peptidoglycan-associated lipoprotein LapL
BJD94_20820	Down	-2.264	4.561E-05	Hypothetical protein
BJD94_02535	Down	-2.2437	1.341E-09	Hypothetical protein
BJD94_07085	Down	-2.2347	0.0020064	Asparagine synthetase [glutamine-hydrolyzing]
BJD94_01835	Down	-2.1417	0.0047799	Transcriptional regulator for fatty acid degradation FadR2C GntR family
BJD94_19275	Down	-2.1121	2.694E-18	Permease of the drug/metabolite transporter (DMT) superfamily

BJD94_10970	Down	-2.0684	0.004307	Predicted membrane protein hemolysin III homolog
BJD94_08635	Down	-2.0511	1.448E-08	Transcriptional regulator VpsR
BJD94_02515	Down	-2.0381	0.0419599	Nicotinate phosphoribosyltransferase
BJD94_19515	Down	-2.025	0.003971	Protease-related protein
BJD94_19600	Down	-1.9812	0.0014704	Kinesin-related protein K4
BJD94_06620	Down	-1.899	0.0181113	Cell division trigger factor
BJD94_00220	Down	-1.8749	0.0151325	UPF0325 protein YaeH
BJD94_15025	Down	-1.8569	0.0261202	Glycerol-3-phosphate transporter
BJD94_04080	Down	-1.8119	1.02E-69	Ribosome modulation factor
BJD94_00545	Down	-1.7824	1.867E-11	Flagellin protein FlaA
BJD94_14015	Down	-1.7379	0.0348734	Dihydrolipoamide dehydrogenase of pyruvate dehydrogenase complex
BJD94_09340	Down	-1.696	0.0004314	Probable transcriptional activator for leuABCD operon
BJD94_20120	Down	-1.687	0.022991	N-acetylglutamate synthase
BJD94_04950	Down	-1.6576	0.0150977	Hypothetical protein

BJD94_05300	Down	-1.6432	0.0261664	DNA-binding protein inhibitor Id-2-related protein
BJD94_07175	Down	-1.617	0.0069305	Thioredoxin domain-containing protein EC-YbbN
BJD94_19800	Down	-1.5976	0.0208742	Maltose/maltodextrin ABC transporter2C substrate binding periplasmic protein MalE
BJD94_18485	Down	-1.5862	0.0412228	Transcriptional regulator2C LacI family
BJD94_12770	Down	-1.5791	0.0002399	Hydrogen peroxide-inducible genes activator
BJD94_19360	Down	-1.5407	1.318E-06	2-amino-3-ketobutyrate coenzyme A ligase
BJD94_05155	Down	-1.5219	0.0401804	Cystathionine beta-lyase
BJD94_14710	Down	-1.5034	0.0021026	Glycerol kinase
BJD94_11520	Down	-1.4458	3.484E-22	Universal stress protein A
BJD94_19450	Down	-1.4114	0.040021	RTX toxins and related Ca2+-binding proteins
BJD94_04910	Down	-1.3507	0.0010348	ATPase of the AAA+ class
BJD94_11105	Down	-1.3199	0.0472063	ATP synthase protein I2

BJD94_06380	Down	-1.3113	0.0004144	Predicted metal-dependent hydrolase with the TIM-barrel fold
BJD94_04860	Down	-1.3054	0.0491304	NAD-dependent malic enzyme
BJD94_07435	Down	-1.2438	0.00138	UDP-sugar hydrolase %3B 5'-nucleotidase
BJD94_06595	Down	-1.2166	0.0036139	Peptidyl-prolyl cis-trans isomerase PpiD
BJD94_07115	Down	-1.1132	0.0354037	Chaperone protein HtpG
BJD94_04090	Down	-1.1131	0.0209487	ABC transporter ATP-binding protein uup
BJD94_04120	Down	-1.1044	0.0035731	Membrane alanine aminopeptidase N
BJD94_04710	Down	-1.0831	0.0024223	
BJD94_21755	Down	-1.0628	0.0007897	C4-dicarboxylate transporter DcuB
BJD94_02105	Down	-1.0573	0.0014765	radical activating enzyme
BJD94_00550	Down	-1.0053	0.0021377	Flagellin protein FlaG
	Up	1.0058	5.288E-06	Hypothetical protein
BJD94_09820	Up	1.0203	0.0282601	LSU ribosomal protein L5p (L11e)

BJD94_10115	Up	1.0319	0.0023524	UDP-N-acetylglucosamine 42C6-dehydratase
BJD94_07305	Up	1.0439	0.0020587	Flagellar basal-body rod protein FlgB
BJD94_11320	Up	1.0452	0.0369803	Ketol-acid reductoisomerase
BJD94_05400	Up	1.0523	0.009655	DNA-binding protein H-NS
BJD94_09445	Up	1.1004	0.0318936	LSU ribosomal protein L21p
BJD94_07300	Up	1.1358	0.0165943	Flagellar basal-body rod protein FlgB
BJD94_20350	Up	1.1533	0.0106798	Predicted phosphatases
BJD94_10500	Up	1.1724	0.0002628	UDP-N-acetylglucosamine 42C6-dehydratase
BJD94_16200	Up	1.2085	0.0010037	UPF0265 protein YeeX
BJD94_18405	Up	1.2118	1.417E-05	Glutathione S-transferase
BJD94_13175	Up	1.2471	0.0003498	Predicted ATPase related to phosphate starvation-inducible protein PhoH
BJD94_09310	Up	1.2669	0.0011528	Pyruvate kinase
BJD94_07220	Up	1.2747	1.061E-05	Phosphocarrier protein of PTS system

BJD94_00665	Up	1.2909	0.0066498	Flagellar biosynthesis protein FlhF
BJD94_09585	Up	1.3031	0.0279778	Putative alpha helix protein
BJD94_13755	Up	1.345	0.0002797	Enolase
BJD94_12195	Up	1.3475	0.0008423	Uncharacterized low-complexity protein
BJD94_02530	Up	1.3708	1.448E-08	Integration host factor alpha subunit
BJD94_12290	Up	1.3784	6.156E-10	22C3-bisphosphoglycerate-independent phosphoglycerate mutase
BJD94_12630	Up	1.379	1.121E-07	Triosephosphate isomerase
BJD94_01055	Up	1.3861	7.864E-10	Uridine phosphorylase
BJD94_00580	Up	1.4271	0.001307	Flagellar regulatory protein FleQ
BJD94_13750	Up	1.4471	5.776E-05	CTP synthase
BJD94_03985	Up	1.462	5.325E-05	Universal stress protein E
BJD94_07375	Up	1.4936	9.951E-15	
BJD94_05430	Up	1.5061	0.0005976	Putative lipoprotein precursor
BJD94_01780	Up	1.5263	4.2E-06	Acetate kinase
BJD94_09360	Up	1.5323	0.0321349	2-isopropylmalate synthase

BJD94_07380	Up	1.5529	2.553E-07	CopG protein
BJD94_06875	Up	1.584	0.0060164	UPF0325 protein YaeH
BJD94_07660	Up	1.5906	0.0005992	Lipoate synthase
BJD94_12235	Up	1.5948	4.903E-06	Succinate dehydrogenase flavoprotein subunit
BJD94_12700	Up	1.607	0.013813	LSU ribosomal protein L31p @ LSU ribosomal protein L31p2C zinc-dependent
BJD94_10980	Up	1.6471	0.0037744	Protein yihD
BJD94_00575	Up	1.6941	0.0241956	Flagellar sensor histidine kinase FleS
BJD94_06220	Up	1.6943	1.142E-36	Alcohol dehydrogenase %3B Acetaldehyde dehydrogenase
BJD94_12450	Up	1.7045	5.479E-18	3'-to-5' exoribonuclease RNase R
BJD94_12730	Up	1.7414	0.0288693	Transcriptional regulator2C PadR family *aphA*
BJD94_12665	Up	1.7484	0.0001001	Ribonuclease E inhibitor RraA
BJD94_12175	Up	1.8042	2.374E-07	6-phosphofructokinase
BJD94_14720	Up	1.8057	0.0326694	22C32C42C5- tetrahydropyridine-22C6-

				dicarboxylate N-succinyltransferase
BJD94_08750	Up	1.8356	2.715E-08	Anaerobic aerobic respiration control protein arcA
BJD94_11225	Up	1.8437	0.0005701	NADH dehydrogenase subunit II-related protein
BJD94_00100	Up	1.9029	0.0016516	Na(+)-translocating NADH-quinone reductase subunit F
BJD94_00805	Up	1.9081	0.0005814	Phosphohistidine phosphatase SixA
BJD94_01050	Up	1.9129	0.0163186	Lead2C cadmium2C zinc and mercury transporting ATPase %3B Copper-translocating P-type ATPase copA
BJD94_12875	Up	1.9217	2.125E-08	Glucose-6-phosphate isomerase
BJD94_18845	Up	1.922	0.0009791	NAD-dependent formate dehydrogenase alpha subunit fdhA
BJD94_05090	Up	1.9572	6.513E-13	Heat shock protein HslJ
BJD94_02165	Up	1.961	2.633E-05	Hypothetical protein
BJD94_21460	Up	1.983	0.0177407	Uncharacterized paraquat-inducible protein B

BJD94_16540	Up	2.0361	3.504E-09	Lipoprotein
BJD94_04705	Up	2.113	0.0163186	Peptidase2C M20A family
BJD94_08370	Up	2.1216	5.501E-06	Phosphate regulon transcriptional regulatory protein PhoB (SphR)
BJD94_09830	Up	2.1778	2.926E-05	LSU ribosomal protein L14p (L23e)
BJD94_17640	Up	2.1821	5.062E-10	Hypothetical protein
BJD94_12250	Up	2.2123	0.0418079	Transcriptional regulator TetR family
BJD94_07895	Up	2.219	0.0005739	Aminoacyl-histidine dipeptidase (Peptidase D)
BJD94_06870	Up	2.2302	3.066E-05	Starvation lipoprotein Slp paralog
BJD94_05435	Up	2.2395	7.273E-11	Hypothetical periplasmic protein
BJD94_05440	Up	2.2587	4.132E-06	Hypothetical protein
BJD94_04635	Up	2.2792	9.7E-27	Catalase / Peroxidase
BJD94_08685	Up	2.2816	0.0010885	UPF0246 protein YaaA
BJD94_07415	Up	2.2856	0.0261664	Ribosomal large subunit pseudouridine synthase F
BJD94_03600	Up	2.2883	0.0005204	Hypothetical protein

BJD94_05270	Up	2.2935	6.156E-10	Trimethylamine-N-oxide reductase
BJD94_11345	Up	2.2995	0.0104787	ATP-dependent DNA helicase Rep
BJD94_20925	Up	2.391	0.0314181	Acetate kinase
BJD94_16315	Up	2.4207	0.0016353	Hypothetical protein
BJD94_15840	Up	2.4581	4.175E-06	Hypothetical protein
BJD94_05450	Up	2.5368	2.541E-07	Hypothetical protein
BJD94_07945	Up	2.6072	0.00138	S-formylglutathione hydrolase fghA
BJD94_09625	Up	2.6623	6.43E-08	Hypothetical protein
BJD94_05445	Up	2.6986	5.169E-15	Cyclopropane-fatty-acyl-phospholipid synthase cdfA
BJD94_19310	Up	2.7082	0.0026224	Catalase
BJD94_05455	Up	2.7183	1.684E-14	Amine oxidase
BJD94_02155	Up	2.7681	1.852E-05	Response regulator containing a CheY-like receiver domain and an HD-GYP domain
BJD94_05465	Up	2.7943	2.717E-18	Putative transcriptional regulator

BJD94_17070	Up	2.9186	0.0045962	Membrane fusion protein of RND family multidrug efflux pump
BJD94_05275	Up	2.9192	0.0022438	Cytochrome c-type protein NapC
BJD94_06890	Up	2.935	4.148E-11	Hypothetical protein
BJD94_09470	Up	2.9439	7.169E-05	TRAP-type uncharacterized transport system2C fused permease component
BJD94_19825	Up	2.9512	0.0401804	Permease of the drug/metabolite transporter (DMT) superfamily
BJD94_15790	Up	3.1029	0.040021	AzlC family protein :branched-chain amino acid ABC transporter permease
BJD94_00510	Up	3.1711	0.0025818	Hypothetical protein
BJD94_08700	Up	3.2151	0.0072679	Hypothetical protein :Hemerythrin-like;
BJD94_15460	Up	3.3385	6.211E-36	Alkyl hydroperoxide reductase protein C ahpC
BJD94_15050	Up	3.537	0.0454705	Anaerobic C4-dicarboxylate transporter DcuC
BJD94_05460	Up	3.5701	4.39E-08	short-chain dehydrogenase/reductase family

BJD94_03280	Up	3.6379	0.031456	Hypothetical protein
BJD94_08630	Up	3.6787	0.0039641	Hypothetical protein purA
BJD94_16325	Up	3.7405	0.0005204	Adenylosuccinate synthetase
BJD94_08715	Up	3.8163	3.65E-108	Pyruvate formate-lyase tdcE or grcA
BJD94_07940	Up	4.215	5.301E-33	S-(hydroxymethyl) glutathione dehydrogenase frmA
BJD94_01215	Up	4.2489	0	Pyruvate formate-lyase
BJD94_11680	Up	4.7018	0.0070725	Hypothetical protein :
BJD94_05490	Up	4.7289	0.000647	Biotin synthase bioB
BJD94_05480	Up	4.7604	0.0059387	Biotin synthesis protein BioC bioC
BJD94_00530	Up	5.2078	0.0012895	Predicted dye-decolorizing peroxidase (DyP) subgroup tyrA
BJD94_15615	Up	5.2368	0.0010441	Tripeptide aminopeptidase
BJD94_19655	Up	6.3833	2.541E-05	periplasmic phosphate-binding protein PstS
BJD94_08580	Up	6.597	1.027E-05	Sodium-dependent phosphate transporter nptA
BJD94_05475	Up	6.6448	7.661E-06	Dethiobiotin synthetase bioD

BJD94_18580	Up	6.6752	1.523E-06	Beta-glucosidase
BJD94_05120	Up	7.4182	5.596E-11	Hydroxylamine reductase hcP
BJD94_16465	Up	7.8656	6.173E-09	Hypothetical protein

Table S2. Primers used in this study

Primer name	Sequence (5’-3’)
pCVD442_forward	GTTATGCCGCTCAATTCGCT
pCVD442_reverse	CGCTGTCGTTCTCAAAATCG
ArcA_Gm_F	CAAATTTAGGTACCGCCACGAATTAGCTTCAAAAGCGCTCTG
ArcA_Gm_R	TTTGTTTATGCGGTGAGCCGAATTGGGGATCTTGAAGTTCCTATTCC
*arcA*_Ups_pCVD442_fwd-NEB	CGTGCCAGGTGCTGCCTCAGATTCACGGCCCCAGTGATTCTTTC
*arcA*_Ups_rev-NEB	TCGTTGCCGGCCTGGCGGTACCTAAATTTGTG
*arcA*_Downs_fwd-NEB	ACAACAGGCTGCGCTCACCGCATAAACAAAAAAAG
*arcA*_Downs_pCVD442_rev-NEB	ACGCAGCGAATTGAGCGGCATAACCTTCCAATATGATTGCAAAAGATC
*arcA*_detectF	GGCAGATTGAGTAAAGTGAC
*arcA*_detectR	CAGCCACGTAGAGAATCATA
*arcA*_internalR	GCATAACACTGCTGCTCATA
